# Case Report: A 13-year-old adolescent diagnosed as malignant phyllodes tumor combined with rhabdomyosarcoma differentiation

**DOI:** 10.3389/fonc.2023.1233208

**Published:** 2023-09-27

**Authors:** Jie Lian, Lu Gao, Ru Yao, Yidong Zhou, Qiang Sun

**Affiliations:** Department of Breast Surgery, Peking Union Medical College Hospital, Chinese Academy of Medical Sciences and Peking Union Medical College, Beijing, China

**Keywords:** phyllodes tumor, rhabdomyosarcoma, treatment strategy, heterogeneous differentiation, pediatric sarcoma

## Abstract

Phyllodes tumor (PT) is an infrequent type of breast neoplasm, constituting a mere 0.5%–1.5% of the entirety of breast tumors. The malignant phyllodes tumor (MPT) comprises only 15% of all phyllodes tumors, and its transformation into rhabdomyosarcoma (RMS) is exceedingly rare in clinical practice. Given its insensitivity to chemotherapy and radiotherapy, treatment options for MPT patients are limited, leaving complete surgical resection as the only option. Therefore, it is imperative to investigate the effective utilization of the heterogeneous differentiation characteristics of MPT to expand treatment alternatives for these patients. In this case report, we represent a 13-year-old adolescent diagnosed with giant breast MPT with RMS differentiation and pulmonary metastasis. The initial step in the treatment process involved radical surgical resection, followed by the administration of four cycles of VDC/IC chemotherapy, which is widely recognized as the standard chemotherapy for RMS. Regrettably, the delay in initiating chemotherapy resulted in minimal observable changes in the size of the pulmonary metastatic nodule. Additionally, a comprehensive literature review on the characterization of MPT with heterogeneous differentiation was conducted to enhance comprehension of the diagnosis and treatment of this uncommon disease in clinical practice. Meanwhile, this case also reminds the doctors that when we diagnose a patient as MPT, it is crucial to consider its heterogenous nature and promptly initiate adjuvant treatment. By targeting the differentiation element of MPT, it becomes feasible to overcome the previously perceived limitation of surgical intervention as the sole treatment option.

## Introduction

Phyllodes tumor (PT), classified as the fibroepithelial tumor of breast composing of epithelial and stromal elements, is a rare kind of pathological subtype in the clinical practice (1). The World Health Organization (WHO) has recently revised the classification of phyllodes tumors based on their histopathological characteristics. The PT can be divided into benign PT, borderline PT, and malignant PT (MPT), where MPT accounts for 15% in all PT (2). (MPT) is characterized by histopathological features such as stromal hypercellularity, atypia, increased mitoses of ≥10/10 high-power fields (HPFs), permeative tumor borders, and stromal overgrowth (3, 4). The stromal components of MPT exhibit heterogeneity and have the potential to transform into rhabdomyosarcoma (RMS), liposarcoma, and osteosarcoma (5). The absence of effective therapeutic interventions, coupled with the propensity for early distant metastasis and frequent recurrence, presents a formidable challenge in clinical practice. The average age of diagnosis for PT is 40–45 years, which is comparatively younger than the typical age range (2, 6). Previously, there have been reports of adolescent patients diagnosed with PT. However, there is a scarcity of reports on adolescent patients diagnosed with MPT with RMS differentiation. In this study, we present a case of a 13-year-old adolescent diagnosed with the rare giant MPT of the breast combined with RMS differentiation. Additionally, we provide a comprehensive summary of the clinical characteristics of such patients, aiming to serve as a valuable resource for the diagnosis and treatment of similar cases.

## Case presentation

**Figure 1:**
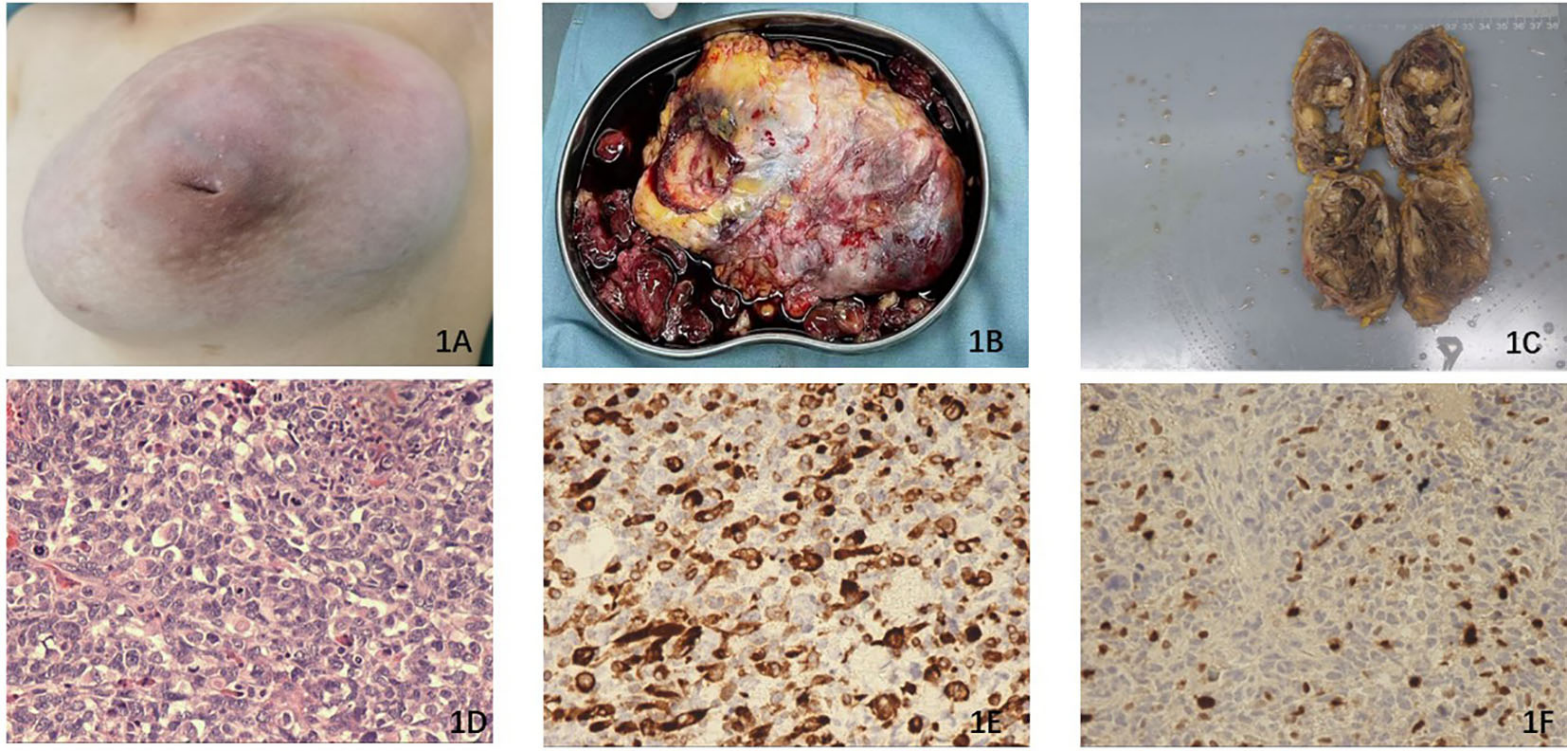
**(A)** The left breast exhibited nipple inversion and is filled with a large combined mass. **(B)** The broken surgical piece of the mass. **(C)** The surgically removed mass is longitudinally incised and measures 13.0 cm ×10.0 cm × 9.0 cm in diameter. **(D)** The histopathological section (hematoxylin and eosin staining) of the tissue from the surgical piece. **(E)** Immunohistochemistry results revealed the expression of Desmin, which is considered a characteristic feature of RMS. **(F)** Immunohistochemistry results demonstrated the expression of Myogenin.

A 13-year-old female patient was initially referred to our hospital due to the presence of a large mass in her left breast, accompanied by nipple inversion (**Figure 1A**). She reported that the mass had been in existence for a duration of 3 years; however, initially, the only symptom observed was asymmetry in the size of her bilateral mammary glands, which did not receive significant attention. In March of this year, the mass began to exhibit progressive growth, with an accelerated rate of growth observed since July. A breast ultrasound examination was carried out at a separate medical institution, which identified a substantial mass measuring 13.6 cm ×10.9 cm ×7.0 cm with an irregular contour, completely occupying and substituting the tissue of the left breast. The mass exhibited progressive growth starting in March of this year, with an accelerated rate of growth observed since July. Subsequently, a core biopsy was conducted to assess the pathological characteristics of the mass. The findings from the core biopsy revealed that the mass was a fibroepithelial tumor exhibiting atypia and necrosis, with the possibility of being classified as a borderline type.

After being referred to our hospital, a repeat breast ultrasound evaluation was performed. The results indicated the presence of a 13.1 cm ×11.8 cm ×8.6 cm mixed-echo mass in the left breast, accompanied by a dot strip blood flow signal. Additionally, the normal mammary gland was significantly compressed. The mammographic diagnosis confirmed that the mass occupied the entire left breast.

The physical examination indicated that the mass exhibited characteristics of both cystic and solid nature, displaying limited activity. No pertinent family history pertaining to the breast tumor was reported. Given the suspicion surrounding the pathological attributes of the mass, coupled with the patient’s young age, a comprehensive excision of the mass was performed (**Figures 1B, C**). Subsequently, the excised mass was sent to the pathology department for identification of its pathological subtype. Following the surgery, the patient experienced a smooth recovery and was discharged to her residence after 3 days. The pathological findings ultimately confirmed that the mass was a combination of MPT with heterogenous differentiation, specifically rhabdomyosarcoma, as depicted in **Figure 1D**. Immunohistochemistry results revealed weakly positive staining for AE1/AE3, positive staining for Desmin (**Figure 1E**), and positive staining for Myogenin (**Figure 1F**). Additionally, the Ki67 index was determined to be 75%. Desmin and myogenin were identified as useful markers for differential diagnosis (7).

**Figure 2:**
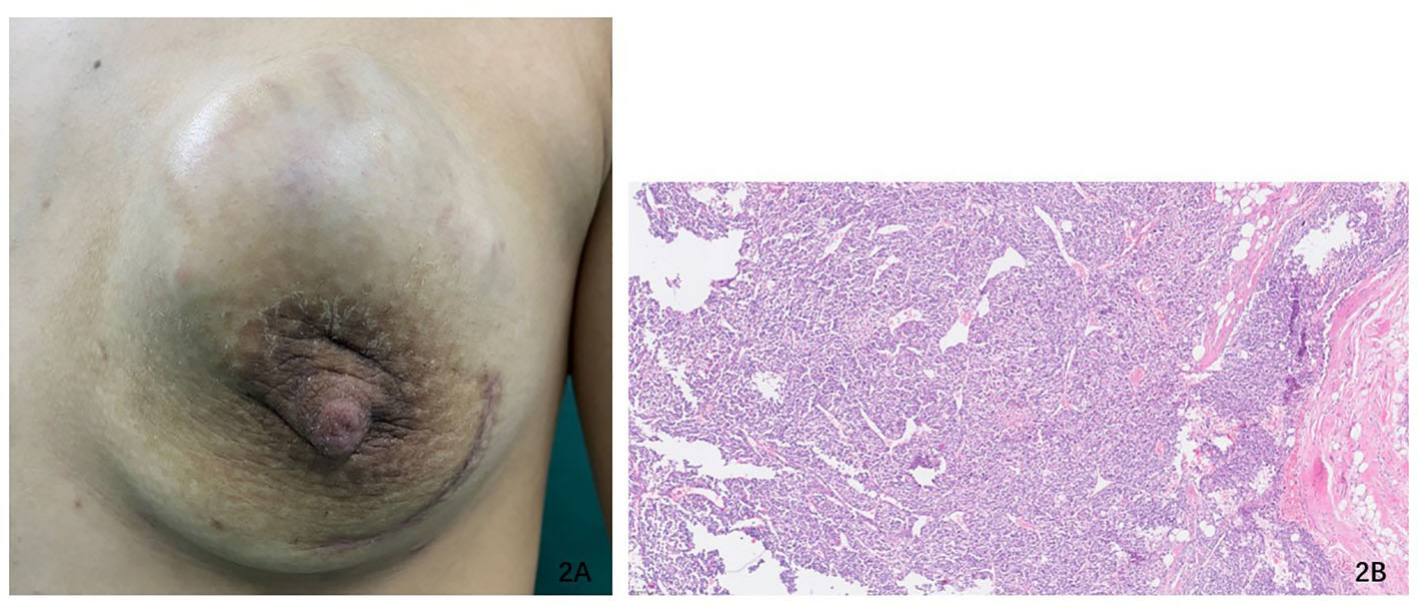
**(A)** The recurrent mass presented a diameter of 5.7 cm × 5.5 cm ×4.5 cm in the left breast within 2 months. **(B)** PT × 40—the histopathological section (hematoxylin and eosin staining) of the tissue from the second surgical piece.

Regrettably, local recurrence occurred rapidly, with the reappearance of the mass in the left breast resembling a ping pong ball and exhibiting aggressive growth on 3 October (**Figure 2A**). Consequently, the patient was re-referred to our hospital for further evaluation. A repeat breast ultrasound was conducted to assess the size and characteristics of the lesion, revealing a 5.7 cm×5.5 cm ×4.5 cm mixed-echo composition with strip blood flow signal. Based on these findings, it was postulated that the possibility of a malignant phyllodes tumor (MPT) was considerable. Additionally, MPTs with a high expression level of Ki-67 (>50%) are more likely to exhibit distant metastasis (8, 9). Considering the rapid recurrence following the previous operation and the high level of Ki-67 expression of the mass, the mastectomy procedure was conducted with the objective of ensuring a maximally negative margin, while the operation area was subjected to warm distilled water immersion to induce hypotonic lysis and subsequent death of tumor cells. The patient exhibited satisfactory recovery and was discharged from the hospital.

Subsequent pathological examination revealed the presence of a heterogenous differential component, specifically rhabdomyosarcoma combined with slice necrosis, consistent with previous findings. Immunohistochemical analysis demonstrated weakly positive expression of SMA and AE1/AE3, positive expression of Desmin and CD34, and an elevated Ki67 index of 85% **Figure 2B**. It indirectly demonstrated that the stroma of the mass had the prominent role in the development of the MPT progression in our patient.

**Figure 3:**
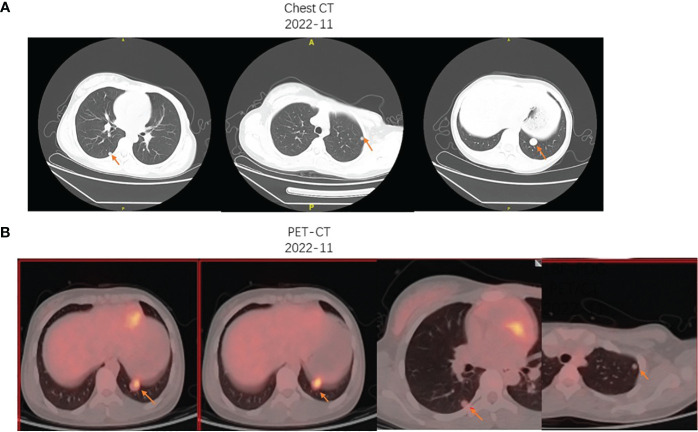
**(A)** The chest CT imaging presented the multiple pulmonary nodules in the right and left lung, with the biggest metastasis measuring nearly 2 cm. **(B)** The 18F-FDG PET/CT showed an increased FDG uptake of the largest left pulmonary nodule (inferior lobe; SUVmax, 3.9) and mild FDG uptake of the multiple pulmonary nodules.

Then, she went to another hospital for further treatment. Unfortunately, about weeks ago, pulmonary nodules were found by the computer tomography (CT) and positron emission tomography/computed tomography (PET/CT) (**Figures 3A, B**), which were suspected to be the lung metastasis. Chemotherapy should be promptly initiated to impede the further progression of the tumor. Consequently, the patient underwent four cycles of chemotherapy, with cycles 1 and 3 consisting of vincristine 2 mg/m^2^, doxorubicin 30 mg/m^2^, and cyclophosphamide 500 mg/m^2^, and cycles 2 and 4 comprising ifosfamide 100 mg/m^2^ and etoposide 1.8 g/m^2^. Every cycle was separated by 1 month. However, following two cycles of chemotherapy, the size of the suspected lung metastasis decreased from 2 cm to 1.7 cm, indicating a partial response, although the overall efficacy of the chemotherapy was not satisfactory. Following the subsequent evaluation, the patient had resection of the lung nodule and undergo the subsequent cycles of chemotherapy.

## Discussion

PT is a rare form of breast neoplasm, comprising 0.5%–1.5% of all cases, while MPT accounts for only 10%–15% of all PTs (8, 10). It constitutes complex mammary fibroepithelial lesions and can be graded as begin, broadline, and malignant. Although benign tumors are the most common, some PTs have the potential to locally recur and progress to sarcoma, as exemplified in the case we present. Notably, the incidence and recurrence rates of PT are higher among Asian patients compared to those in Western countries (11). The feature in our case is that the patient is diagnosed with MPT combined with RMS differentiation. It is worth mentioning that MPT can be mistakenly identified as primary breast sarcoma due to the presence of heterologous sarcomatous differentiation in the stromal tissue. A key distinction between MPT with sarcomatous differentiation and primary sarcoma is the absence of an epithelial component in the latter. In order to provide a comprehensive understanding, we have conducted a review of the clinical characteristics of PT with heterogeneous elements, which are presented in **Table 1** (12–19). In our reported case, the predominant recurrence element is rhabdomyosarcoma, while the presence of epithelium in MPT is scarcely observed. This observation aligns with the characteristic of MPT, which involves uncontrolled growth of the stroma and epithelial outgrowth. Previous studies speculated that the overexpression of IGF-II and c-myc would drive the invasive stromal proliferation and sarcomatous differentiation, while c-kit expression was associated with poor prognosis and could be designed as a therapeutic target (20). Recently, Ahmed et al. demonstrated that the expression level of cancer stem markers in the stroma of MPT were negatively correlated with the overall survival of MPT patients, and the dysregulation of epithelial–mesenchymal transition may fuel the aggressive behavior of the stroma in MPT (4).

**Table 1:** The clinical characteristics of PT with heterogeneous elements previously reported.

No.	Reference	Sex/ Age	Size	Malignant/ Begin/ Broadline	Heterogenous element	IHC	Metastatic interval	Metastatic site	Therapeutic strategy	Prognosis until reported
1	Barnes, L. et al. 1978 (12)	F/45	10~12 cm	Malignant	rhabdomyosarcoma	NA	2 years	lung, brain	1.radical mastectomy for the breast mass 2.the entire left resection for the lung metastasis 3. irradiation for the brain metastasis	died 2.5 years after the first breast mass radical mastectomy
2	Guerrero, M.A et al. 2003 (13)	F/96	17 x 17 x 8 cm	Malignant	liposarcoma, leiomyosarcoma, rhabdomyosarcoma, malignant fibrous histiocytoma	NA	NA	NA	wide excision	died 10 months after the surgery
3	Tsubochi, Sato et al. 2004 (14)	F/54	9 x 7 x 6 cm	Malignant	osteosarcoma	vimentin (+), cytokeratin (+), S-100(+), CD34(+), and HER2(+), P53(-)	1 year	lung	subcutaneous mastectomy	NA
4	Vergine, Guy et al. 2015 (15)	F/71	2.5 cm	Malignant	Melanoma	Melan A (+), pan-melanoma (+), S100(+), HMB45(+), CD34(+), SMA (+), AE1/3(-), P53(-), MIB1(+)	NA	NA	completion mastectomy	without relapse or distant metastasis
5	Narla, Stephen et al. 2018 (16)	F/28	14 x 14 x 8 cm	Malignant	liposarcoma	NA	NA	NA	completion mastectomy	lost to follow-up
6	Jin, Bi et al. 2021 (17)	F/59	5.5 x 4.0 x 3.5 cm	Malignant	osteosarcoma	SMA (+), SATB2(+), Ki67(40%), Ckpan (-)	NA	NA	wide local excision	without relapse or distant metastasis
7	Tu He Ta Mi Shi, Wang et al. 2021 (18)	F/52	8 x 6 x 5.5 cm	Malignant	mixed liposarcoma (myxoid liposarcoma and pleomorphic liposarcoma	AE1/3(+), vimentin (+), S-100(-), Ki67(90%), E-cadherin (-), p63(-)	NA	NA	radical mastectomy	without relapse or distant metastasis
8	Han, Liu et al. 2022 (19)	F/69	4.3 x 4.1 x 3.3cm	Malignant	rhabdomyosarcoma	MyoD1(+), myogenin(+), desmin(+), α-SMA(+), Ki67(63%)	NA	NA	radical mastectomy, VAC therapy (vincristine, actinomycin D, cyclophosphamide)	without relapse or distant metastasis

NA, not mentioned in the reported article.

Previous studies revealed that if the RMS was the major component in the MPT, the difference in clinical presentation and therapeutic strategies may not be so obvious between the MPT with RMS differentiation and the primary RMS. Therefore, it is imperative to comprehensively consider the clinical features of both MPT and rhabdomyosarcoma in order to develop appropriate management strategies for our reported patient (21). RMS is an infrequent non-epithelial tumor in clinical practice, primarily affecting children and comprising approximately 50% of soft tissue tumors. However, its occurrence in breast malignant tumors is exceedingly rare, accounting for <1% of total RMS cases (22, 23).

The clinical presentations of MPT and RMS are similar. Both of them may represent with the rapidly increased painless mass (24), potentially accompanied by symptoms resulting from the mass’ impact on neighboring organs and neurovascular structures (25). In reality, the majority of patients may initially disregard the mass until it undergoes rapid growth within a brief timeframe, similar to the patient that we present, who initially only observed the asymmetry of their bilateral mammary glands. Additionally, the infrequent symptom of hypoglycemia resulting from the elevated expression level of IGF-2 in the tumor tissue has been previously documented (26). The physical examination findings in our patient encompass nipple inversion, a mass exhibiting both cystic and solid components with limited activity. Other symptoms, such as skin ulceration, invasion of the chest wall, and bloody nipple discharge, have also been sporadically reported (6). The occurrence of quick local recurrence (LR) is an additional characteristic observed in our case. In comparison to begin PT, the frequency of LR was higher in MPT (8% vs. 18%) (27). Multicenter investigations have indicated that positive margin serves as an independent risk factor for LR, while other risk factors encompass breast-conserving surgery, negative margins measuring <1 cm, tumor size ≥5 cm, mitoses, infiltrating tumor border, moderate/severe stromal cellularity, severe stromal atypia, severe stromal overgrowth, and tumor necrosis (27–29). Unfortunately, the patient that we report in this case had several risk factors for recurrence including the omission of the chemotherapy after the first surgery, acceptance of breast-conserving surgery, tumor size ≥ 5 cm, and combination of tumor necrosis. Therefore, the recurrence happened just 2 months after the surgery. The phenomenon that the recurrence component is mainly the RMS demonstrated that the aggressive behavior of MPT may be caused by the stroma. Furthermore, hematogenous metastasis happens more frequently than lymph node metastasis for MPT, and previously reported metastatic sites for MPT included lung, liver, adrenal, brain, bone, duodenum, heart, orbit, and ovarian (2, 8, 30). Meanwhile, the lung is also the common metastatic site for RMS, thus considering the lung nodule in our patient as the metastatic MPT and initiating the adjuvant chemotherapy immediately were reasonable (31).

The gold standard for the diagnosis of MPT with heterogenous differentiation still relies on pathology. Core needle biopsy is a valuable diagnostic tool, and the pathology should include stromal hypercellularity, atypia, increased mitoses of ≥10/10 HPFs, permeative tumor borders, and stromal overgrowth (3). In addition, the existence of heterologous sarcomatous differentiation, such as RMS differentiation in our cases, will make PTs classified as MPT regardless of other pathological features (32). Rhabdomyoblasts cells with atypia such as ribbon, tadpole-like, oval shape, or undifferentiated rhabdomyoblasts with scant cytoplasm are crucial for the diagnosis of RMS (33). Furthermore, RMS cells also represent skeletal muscle gene products such as myosin, desmin, myoglobin, and MYOD1 immunohistochemically (25). However, confined by the limitation of the tissue size obtained from the core needle biopsy, the diagnosis may not be accurate regardless of MPT or RMS, while sometimes, histopathological examination after complete removal of the tumor was indispensable just as in the case we reported. For example, delayed diagnosis for RMS happens frequently, as fibroadenoma and mastopathy may take up the majority of the mass and interfere the judgement of the pathologist (34). As for non-invasive examination, in our case, the difference between ultrasound outcomes before the first surgery and after the recurrence was not significant. In fact, as for MPT with RMS differentiation, imaging examination can facilitate in locating the mass, evaluating the invasion of the border, assessing the distant metastasis, and stratifying the risk level, but may be not conductive to distinguish different breast tumors. However, we still recommended that the patient in our case receives the ultrasound examination monthly after the second surgery to detect the recurrence as early as possible.

As we mentioned before, the therapeutic strategies should take both the MPT and RMS into consideration, especially for RMS, as the main recurrence component identified in our patient. For MPT, no systemic standard therapy strategies have been established, and the National Comprehensive Cancer Network (NCCN) guidelines recommend the complete surgical resection with at least 1-cm margins without sentinel lymph node biopsy for the treatment of MPT. Surgeons may more likely perform breast-conservation operation instead of mastectomy for pediatric MPT patients considering their quality of life in the future (35, 36). However, as previously stated, breast conservation is a risk factor for the LR, and the patient receiving only lumpectomy combined with other risk factors that we report in this case recurred within 2 months after the surgery. The use of adjuvant therapy for MPT has also been explored, but standard pathological data and prospective, multicentric chemotherapy, or radiotherapy studies are needed to improve the overall MPT survival (37). Alkylating-agent-based chemotherapy or the combination of nab-paclitaxel, cisplatin, and liposomal doxorubicin chemotherapy with radiotherapy have been reported to be an effective option for metastatic MPT (38, 39). Adjuvant radiation could reduce the LR for MPT patients receiving extensive local resection but not benefit the MPT patients accepting mastectomy (40).

Different from the dilemma of MPT, which mainly relied on the surgery, it has long been demonstrated that the treatment for RMS is multimodality. Surgery still takes the dominant role in the whole therapeutic strategy. Apart from surgery, RMS is sensitive to cyto-toxic chemotherapy and radiotherapy (25). Vincristine, dactinomycin, and alkylating agents are mainstream drugs for the treatment of the RMS, with an expectation of at least 40% improvement of the survival rate compared with the surgery alone (41, 42). However, chemotherapy should be cautious to be applied for adolescent patients, as alkylating agent chemotherapy will cause the ovarian failure and result in infertility (43). Therefore, Mance et al. harvested and frozon the ova of a 17-year-old patient diagnosed as primary breast RMS before the initiation of adjuvant chemotherapy (44). In addition, cisplatin chemotherapy-related hearing loss was also reported and should be paid attention to in the clinical practice (45). More robust evidence has demonstrated that the use of radiotherapy can improve the long-term prognosis for RMS patients. Radiotherapy is recommended to initiate after 12 weeks of chemotherapy (four cycles), and the overall assessment is necessary. The dose of radiotherapy depends on several factors including the stage, the risk group, the site of RMS, the histology group, the degree of surgery, and the addition of the chemotherapy (46–48). Delay or omission of radiotherapy may increase the risk of recurrence (42). Europe RMS 2005 study revealed that in combination of radiotherapy, the improvement of approximately 10% in 3-year event-free survival (EFS) was observed for high-risk RMS patients, while the 3-year EFS could be improved from 39% to 56% for very high-risk RMS patients (49). The RMS is sensitive to the ionizing radiation, but the adverse effect brought by radiotherapy is still controversial. Radiotherapy-related adverse events include the secondary malignancy, joint stiffness, facial growth retardation, neuroendocrine dysfunction, and cognitive sequelae (25, 45). To reduce the adverse events brought by traditional radiotherapy, researchers are focusing on the proton radiotherapy. Ladra et al. designed a phase II multicenter clinical research and revealed that proton therapy can reduce the irradiation dose, which decreased the acute toxicity but with the same rate of local disease control (47).A multicenter clinical research in Japan treating children with 36–60 GyE (median, 50.4 GyE) irradiation dose also demonstrated that the proton radiotherapy could achieve the same short-term effect with fewer adverse events compared to photon radiotherapy (48).

For metastatic RMS patients, radiotherapy is still disputed, as the authoritative classification of patient subgroups that can benefit more from the radiotherapy than others are absent and myelosuppression caused by radiotherapy may restrict the effect of chemotherapy (49). However, for patients with one or more lung metastases, whole lung radiotherapy is recommended (50). Compared with other metastatic sites, pulmonary metastatic nodules are more likely to benefit from the radiotherapy. A retrospective review revealed that the pulmonary local control could be improved from 10% to 56% by applying the radiotherapy, and the 5-year-progression-free survival (PFS) for lung metastasis was 29% compared with other types of metastases, which was 7% (51).

The chemotherapy strategy for our patient was the four cycles of vincristine–doxorubicin–cyclophosphamide/ifosfamide–etoposide (VDC/IE) chemotherapy, which is the main chemotherapy strategy for the treatment of Ewing sarcoma family of tumors (52). Furthermore, VDC/IE can be used for intermediate-risk RMS, and GOC ARST0431 revealed that compressing the dosing interval to allow the patients to receive the maximum amount of effective agents at a short period of time in combination with radiotherapy sensitizers can improve 3-year EFS of 69% for metastatic RMS compared with previous RMS therapeutic research. This improvement was achieved without a concomitant rise in the incidence of adverse reactions, thereby surpassing the outcomes of prior therapeutic investigations in RMS (53).

## Conclusion

In this study, we present a case of an adolescent patient who was diagnosed with MPT with RMS differentiation, contributing novel perspectives on treatment approaches for these uncommon diseases. Pathology remains crucial in the diagnosis of MPT, and the integration of imaging examinations can aid in assessing the severity and monitoring the occurrence of relapse and distant metastases. The heterogeneous nature of MPT is of utmost importance in clinical practice, as it can significantly influence disease progression and guide therapeutic decision-making. As in the patient that we report in this case, both the biological feature of MPT and RMS should be taken into consideration for the clinical decision-making. Surgery with negative margin is critical to reduce the recurrence or metastasis and improve the overall survival for both the MPT and RMS patient. Adjuvant chemotherapy and radiotherapy are controversial for pure PT patients, but robust evidence has revealed that RMS patients will benefit a lot from such treatments. It is regrettable that the patient did not promptly receive adjuvant chemotherapy upon initial diagnosis of MPT with RMS differentiation. This serves as a reminder to medical professionals that MPT patients with RMS differentiation can also benefit from the multimodal treatment typically administered to primary RMS patients.

## Data availability statement

The raw data supporting the conclusions of this article will be made available by the authors, without undue reservation.

## Ethics statement

Ethical approval was not required for the study involving human samples in accordance with the local legislation and institutional requirements. Written informed consent for participation in this study was provided by the participants’ legal guardians/next of kin. Written informed consent was obtained from the participant's legal guardian for the publication of this case report and any identifiable material contained.

## Author contributions

YZ designed the idea of the article. JL, YZ, and RY collected the patient’s clinical and pathological data. JL is the major contributor in writing the first draft of the manuscript. YZ and LG revised the manuscript. All authors contributed to the article and approved the submitted version.
